# Redox-Based Inactivation of Cysteine Cathepsins by Compounds Containing the 4-Aminophenol Moiety

**DOI:** 10.1371/journal.pone.0027197

**Published:** 2011-11-04

**Authors:** Bojana Mirković, Izidor Sosič, Stanislav Gobec, Janko Kos

**Affiliations:** 1 Department of Pharmaceutical Biology, Faculty of Pharmacy, University of Ljubljana, Ljubljana, Slovenia; 2 Department of Medicinal Chemistry, Faculty of Pharmacy, University of Ljubljana, Ljubljana, Slovenia; 3 Department of Biotechnology, Jožef Stefan Institute, Ljubljana, Slovenia; Stanford University, United States of America

## Abstract

**Background:**

Redox cycling compounds have been reported to cause false positive inhibition of proteases in drug discovery studies. This kind of false positives can lead to unusually high hit rates in high-throughput screening campaigns and require further analysis to distinguish true from false positive hits. Such follow-up studies are both time and resource consuming.

**Methods and Findings:**

In this study we show that 5-aminoquinoline-8-ol is a time-dependent inactivator of cathepsin B with a k_inact_/K_I_ of 36.7±13.6 M^−1^s^−1^ using enzyme kinetics. 5-Aminoquinoline-8-ol inhibited cathepsins H, L and B in the same concentration range, implying a non-specific mechanism of inhibition. Further analogues, 4-aminonaphthalene-1-ol and 4-aminophenol, also displayed time-dependent inhibition of cathepsin B with k_inact_/K_I_ values of 406.4±10.8 and 36.5±1.3 M^−1^s^−1^. No inactivation occurred in the absence of either the amino or the hydroxyl group, suggesting that the 4-aminophenol moiety is a prerequisite for enzyme inactivation. Induction of redox oxygen species (ROS) by 4-aminophenols in various redox environments was determined by the fluorescent probe 2′,7′-dichlorodihydrofluorescein diacetate. Addition of catalase to the assay buffer significantly abrogated the ROS signal, indicating that H_2_O_2_ is a component of the ROS induced by 4-aminophenols. Furthermore, using mass spectrometry, active site probe DCG-04 and isoelectric focusing we show that redox inactivation of cysteine cathepsins by 5-aminoquinoline-8-ol is active site directed and leads to the formation of sulfinic acid.

**Conclusions:**

In this study we report that compounds containing the 4-aminophenol moiety inactivate cysteine cathepsins through a redox-based mechanism and are thus likely to cause false positive hits in the screening assays for cysteine proteases.

## Introduction

Cathepsins are lysosomal cysteine proteases belonging to the papain-subfamily C1A of the clan CA of cysteine proteases [1]. The group comprises 11 cathepsins (cathepsins B, C, F, H, K, L, O, S, V, W and X) which act predominantly as endopeptidases and are for the most part located intracellularly in endolysosomal vesicles [2]. For this reason it was long believed that their primary function was protein turnover within lysosomes [3]. However, it was later discovered that individual cathepsins could also be associated with more specific functions. For example, cathepsin K is abundant in osteoclasts, where it plays a vital role in the resorption and remodeling of bone [4], and cathepsin S has been implicated in major histocompatibility class II antigen presentation [5]. However, for several cathepsins, dysregulation at the protein, activity and localization levels can lead to numerous pathologies. One of the best studied examples is the causative role of cathepsin B in malignant diseases where it was shown to be involved in tumor formation, growth and invasion, as well as to participate in angiogenesis [2].

Much is now known about the cysteine cathepsins. Their crystal structures, physiological and pathological involvement as well as their regulatory mechanisms constitute them as attractive targets for drug discovery [6], [7]. Cathepsins K, S and B have been validated as effective drug targets in osteoporosis, immune diseases and cancer, respectively [2], [6], [7]. Several drug discovery strategies have been employed in the search for cathepsin inhibitors, such as isolation, characterization [8], [9] and evaluation of compounds of natural origin [10], virtual screening of large libraries of small molecules [11], fragment-based screening [12] and high-throughput screening of large compound libraries [13], to name a few. However, such approaches can often result in nonspecific false positives, characterized by steep dose-response curves, lack of clear structure-activity relationships and high sensitivity to assay conditions [14]. Several mechanisms have been proposed for these artifacts, including the involvement of chemically reactive molecules, molecules that may interfere with the assay signal, and molecules that form aggregates and cause partial denaturation of the target, manifesting themselves as nonspecific or promiscuous enzyme inhibitors [14], [15].

In the last decade a new mechanism of false positive inhibition has been reported for proteases that possess functional groups susceptible to redox modification [16]–[20]. The molecules causing the artifact were termed redox cycling compounds (RCC) due to the formation of mM concentrations of hydrogen peroxide in the presence of reducing agents used in the assay buffers for high-throughput screening (HTS) [16], [17]. H_2_O_2_ generated by such molecules induces oxidation of accessible cysteine, tryptophan, methionine, histidine, or selenocysteine residues, in this way causing false positive inhibition of several classes of proteases, such as protein tyrosine phosphatases [17], [19], cysteine proteases (cathepsins and caspases) [18], [20], [21] and metalloenzymes [16], [21]. This kind of false positive can lead to unusually high hit rates [18] in HTS campaigns, which require further analysis to distinguish true from false positive hits. Such follow-up studies are both time and resource consuming and, if nonspecific inhibitors are not recognized as such, they may be even deposited in screening databases and flagged active [16], [22].

We identified 5-nitroquinolin-8-ol as a selective and potent inhibitor of cathepsin B using virtual-screening, enzyme kinetics and cell-based assays in a previous study [23]. By verifying the structural requirements for cathepsin B inhibition we have now discovered that the 5-amino analogue, 5-aminoquinolin-8-ol (**1**), displays time-dependent inhibition. The inhibition has been shown to be concentration dependent, nonspecific, irreversible and active site directed, using enzyme kinetics, mass spectrometry, the DCG-04 probe and isoelectric focusing (IEF). Furthermore, the inhibition is shown to depend on reactive oxygen species (ROS) using the fluorescent probe 2′,7′-dichlorodihydrofluorescein diacetate (H_2_DCFDA) and is abolished by catalase. Here we report that compounds containing the 4-aminophenol moiety inactivate cysteine cathepsins through a redox-based mechanism and are thus likely to cause false positive hits in screening assays for inhibition of cysteine proteases.

## Materials and Methods

### Reagents

**Figure 1:**
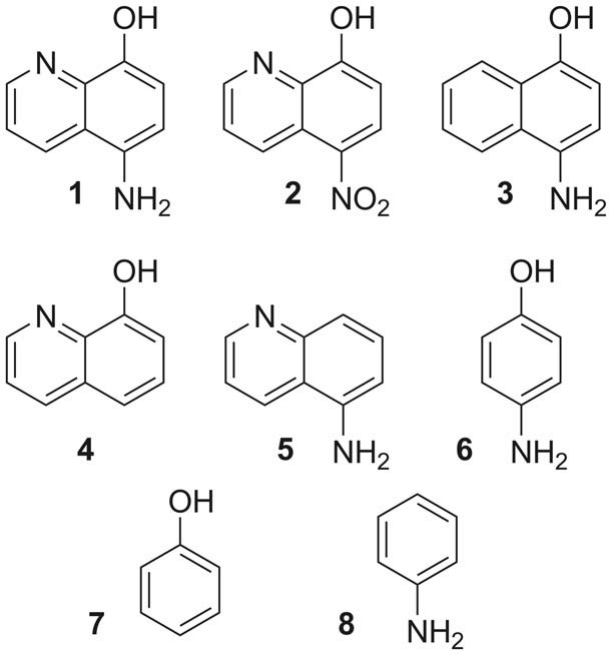
4-Aminophenol derivatives and their analogues used in this study.

5-Aminoquinolin-8-ol (**1**) (95%), 5-nitroquinolin-8-ol (**2**) (96%), 4-aminophenol (**6**) (≥99%), phenol (**7**) (∼ 99%), and aniline (**8**) (≥99.5%) were purchased from Sigma-Aldrich and used without further purification. 4-Aminonaphthalen-1-ol hydrochloride (**3**) (90%), quinolin-8-ol (**4**) (for analysis ACS) and quinolin-5-amine (**5**) (for analysis ACS) were purchased from Acros Organics and used without further purification (Figure 1). Cystine, cysteine and DTT were obtained from Sigma-Aldrich, and H_2_O_2_ from Merck. Irreversible cathepsin B specific inhibitor CA-074 was obtained from Bachem and the active site probe DCG-04 was kindly provided by Dr. Matthew Bogyo.

### Enzymes and assay buffers

Human recombinant cathepsin B was prepared as reported [24]. Human cathepsin H was isolated from human liver [25] and human recombinant cathepsin L was expressed in *E. coli* [26]. Cathepsins B, L and H were assayed in 100 mM phosphate buffer, pH 6.0, 100 mM acetate buffer, pH 5.5 and 100 mM phosphate buffer, pH 6.8, respectively. Each contained 0.1% PEG 8000 (Sigma-Aldrich) and 1.5 mM EDTA (Serva). Activation buffers were prepared by adding reducing agent to the assay buffer to a final concentration of 5 mM. Prior to the assay each enzyme was activated in the activation buffer for 5 min at 37°C.

### Time-dependent inhibition of cathepsins by 4-aminophenols

To evaluate the inhibitory activity of the tested compounds the following substrates were used: Z-Arg-Arg-AMC (Bachem) at 60 µM, Z-Phe-Arg-AMC (Bachem) at 2 µM and Arg-AMC (Biomol) at 10 µM for cathepsins B, L and H, respectively. 5 µl of inhibitor or dimethyl sulfoxide (DMSO) and 5 µl of the appropriate substrate were added to the wells of a black 96-well microplate. The reaction was initiated by adding 90 µl of cathepsins B, L or H at final concentrations of 400 pM, 200 pM or 2.5 nM, respectively. Formation of fluorescent degradation product was monitored continuously at 380 nm±20 nm excitation and 460 nm±10 nm emission on a Tecan Safire2 reader (Tecan) using minimal kinetic interval at 37°C. All kinetic measurements were performed in duplicate. Progress curves obtained with a range of concentrations of inhibitor were fitted to the equation [27] *P  =  v_s_t + (v_i_ − v_s_)(1 − e^−kobst^)/k_obs_*, where *P* denotes the product concentration in relative fluorescence units (RFU) at time *t*; *v_i_* and *v_s_* are the initial and steady-state velocities and *k_obs_* is the apparent first-order rate constant for the establishment of the final steady-state equilibrium. Plots of *k_obs_* values versus inhibitor concentration yielded a linear fit describing a single-step mechanism for the irreversible inactivation of the enzyme, with a slope corresponding to the second-order rate constant *k_inact_/K_I_*.

### Fluorescent probe H_2_DCFDA for ROS detection

H_2_DCFDA (D6883, Sigma) was used as described elsewhere with minor modifications [28]. To hydrolyze the ester functionality of the probe for use in a cell-free assay, H_2_DCFDA was dissolved in DMSO at 1 mM, diluted to 100 µM with 0.01 N NaOH and kept in the dark at room temperature (RT) for 30 min. The reaction was performed in a black 96-well microplate, adding to each well 10 µL of the de-esterified probe solution, 5 µl of DMSO or inhibitor at a final concentration of 500 µM and 85 µl of the activation buffer with different redox agents (redox-free, cystine, cysteine or DTT, each at 5 mM). In a parallel experiment catalase from bovine liver (C9322, Sigma) was added to the activation buffer to a final concentration of 200 U/ml. The reaction was continuously monitored for 15 min using 535 nm excitation and 485 nm emission filters on a Tecan GENios reader (Tecan), with 1 min kinetic interval at 37°C. All measurements were performed in duplicate.

### Active site probe DCG-04

1 µg cathepsin B in the activation buffer with a suitable redox agent at 5 mM was incubated with 200 µM respective inhibitor for 1 h at RT. Samples were labeled with 2 µM DCG-04 for 1 h at RT, separated by 12.5% SDS-PAGE and transferred to a nitrocellulose membrane. The latter was blocked overnight with 0.5% Tween 20 in PBS at 4°C and incubated with 1 µg/ml streptavidin-horseradish peroxidase (Sigma-Aldrich) for 1 h at RT. The membrane was washed five times and the spots on the membrane were visualized with 0.5 mg/ml 3,3′-diaminobenzidine (Sigma-Aldrich) and 0.0005% (v/v) H_2_O_2_.

### Isoelectric focusing

2 µg cathepsin B in the activation buffer with no reducing agent, cystine, cysteine or DTT at 5 mM, respectively was incubated with DMSO or CA-074 at 50 µM for 15 min at RT to prevent redox changes to the active site thiol group. Samples were then incubated with DMSO, compound **1** or H_2_O_2_ at 500 µM or 490 µM, respectively for 5 h at RT. 4 µl of each sample was loaded on the precast isoelectric focusing gel PhastGel IEF (pI range 3.0–9.0) (GE Healthcare) and separated on the Phast System (GE Healthcare) according to the protocol of the manufacturer. After separation, gels were developed on the Phast System using Coomassie blue staining. pIs of individual bands were determined from a calibration curve (Figure S5) obtained using the Broad range pI calibration kit (pH 3.0–10.0) (GE Healthcare).

### Mass spectrometry

Cathepsin B (4.4 µM) was incubated with DMSO or CA-074 (44 µM) in the activation buffer containing 1 mM cysteine for 1 h at RT. Compound **1** (500 µM) or DMSO were added and incubated with cathepsin B for additional 5 h at RT. The mixtures were then dialyzed against water using Microcon Centrifugal Filter Devices YM-10 (Millipore). Samples were prepared by adding an equal volume of acetonitrile containing 0.1% formic acid and analyzed by ESI-MS on an AutoSpec Q instrument (Micromass).

## Results

### Time-dependent irreversible inactivation of cathepsin B by compound 1

**Figure 2:**
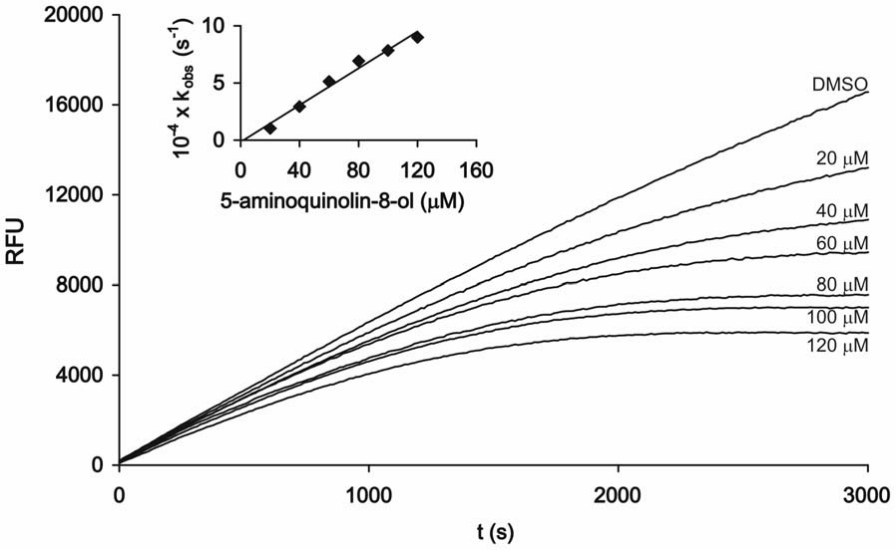
Time-dependence of the inhibition of cathepsin B by compound 1 in the presence of 5 mM DTT. The increasing concentrations of compound **1** were premixed with the fluorogenic substrate Z-Arg-Arg-AMC (60 µM) and the time course of the reaction was measured after the addition of cathepsin B (400 pM) using excitation and emission wavelengths of 380 and 460 nm, respectively. The solid lines represent the raw data which were fitted to the equation *P  =  v_s_t + (v_i_ − v_s_)(1 − e^−kobst^)/k_obs_*, from which the apparent first-order rate constant *k_obs_* was obtained. Insert: Plot of the *k_obs_* values versus concentration of compound **1**. The line represents a linear fit describing a single-step mechanism for the irreversible inactivation of the enzyme, with a slope corresponding to the second-order rate constant *k_inact_/K_I_*.

Under redox-free conditions, compound **1** (Figure 1) potently inhibited cathepsin B in a dose- and time-dependent manner (Figure S1). The activity of cathepsin B decreased in a pseudo-first order process and plotting the apparent first-order rate constant (*k_obs_*) versus inhibitor concentration yielded a linear fit describing a single-step mechanism for irreversible inactivation of the enzyme with a second-order rate constant, *k_inact_/K_I_*, of 36.7±13.6 M^−1^s^−1^ (Table 1). This inactivation rate was decreased approximately 10-fold when reducing agent 5 mM cysteine or 5 mM DTT was introduced into the activation buffer (2.4±0.1 M^−1^s^−1^ and 3.5±1.7 M^−1^s^−1^, respectively) (Table 1). A representative progress curve depicting the time dependence of the inhibition of cathepsin B by compound **1** and plot of k_obs_ versus compound concentration are shown in Figure 2. Cystine did not influence the inactivation rate significantly.

**Table 1:** Second-order rate constants for inactivation of cathepsins B, H and L by compound **1** in the presence of different redox compounds.

k_inact_/K_I_ (M^−1^s^−1^)^a^			
	Cathepsin B	Cathepsin H	Cathepsin L
**Redox-free**	36.7±13.6	35.34±0.75	107.9±10.5
**Cystine**	41.3±16.4	28.0±3.9	41.9±3.6
**Cysteine**	2.4±0.1	37.9±1.0	4.3±3.2
**DTT**	3.5±1.7	7.7±0.45	5.3±1.4

a means ± SD, n≥2. Experimental details are described in the Materials and methods section.

### The inactivation of cathepsin B by compound 1 is nonspecific with regard to other cathepsins

To evaluate whether compound **1** was a cathepsin B specific inhibitor we tested the compound against two related cathepsins, the aminopeptidase cathepsin H and the endopeptidase cathepsin L. Results show that compound **1** inhibited cathepsin H in a manner similar to cathepsin B under non-reducing conditions (redox-free and 5 mM cystine) since the inactivation rates remained almost the same (Table 1). Furthermore, the inactivation rate was also lowered in the presence of the reducing agent DTT, as is the case with cathepsin B. However, when cysteine was added to the activation buffer the inactivation rate was the same as under non-reducing conditions and did not decrease. When inhibitory activity of compound **1** was evaluated on cathepsin L the trend of inactivation was similar to the cathepsin B inactivation – a 10–fold decrease in inhibition after introduction of reducing agents (Table 1). Although the inactivation rate in the redox-free conditions was three times higher than that for cathepsin B, the differences in the inactivation rates between individual cathepsins are too small to conclude that compound **1** is a specific cathepsin B inhibitor.

### Structural requirements for cathepsin inactivation

The structural requirements for the time-dependent irreversible inactivation of cathepsin B by compound **1** were determined by testing its structural analogues (compounds **2**–**8**) (Figure 1) against cathepsin B in two representative redox environments, redox-free and 5 mM cysteine. Results show that the 4-aminophenol moiety is a prerequisite for cathepsin B inactivation. In the redox-free conditions only compounds **1**, **3** and **6** (Figure S1) displayed time-dependent irreversible inhibition. Compound **2** showed reversible inhibition whereas compounds **4**, **5**, **7** and **8** displayed no inhibition at all. Inactivation rates were determined for compounds **1**, **3** and **6** (Table 2). Compound **3** is an 11-fold more potent inhibitor of cathepsin B than compound **1**, whereas compound **6** inhibits cathepsin B with the same potency as compound **1** under the redox-free conditions. Interestingly, when compounds were tested in a reducing environment (5 mM cysteine), only compounds **1** and **3** retained time-dependent irreversible inactivation of cathepsin B (Figure S2). Inactivation rates were determined (Table 2) and compound **3** proved a 42-fold more potent inhibitor of cathepsin B than compound **1** in the presence of 5 mM cysteine. On the other hand, compound **6** did not display the time-dependent irreversible behavior within the time course of the assay in the presence of 5 mM cysteine as it did under the redox-free conditions. Compound **2** retained its reversible inhibition of cathepsin B whereas compounds **4**, **5**, **7** and **8** did not display inhibition of cathepsin B as in the redox-free conditions.

**Table 2:** Second-order rate constants for inactivation of cathepsin B by compounds 3 and 6 and by H_2_O_2_ under different redox states.

k_inact_/K_I_ (M^−1^s^−1^)^a^			
	3	6	H_2_O_2_
**Redox-free**	406.4±10.8	36.5±1.3	43.9±11.1
**Cysteine**	101.1±3.3	NR	NR

a means ± SD, n≥2. NR No reaction observed. Experimental details are described in the Materials and methods section.

### Time-dependent irreversible inactivation of cathepsin B by H_2_O_2_

Since there is no electrophilic warhead in the structure of compound **1** that could explain the irreversible behavior of cathepsin B inhibition, and since RCCs have been reported to be able to generate H_2_O_2_ in HTS buffers [16], we studied the nature of cathepsin B inhibition by H_2_O_2_. In the redox-free environment H_2_O_2_ inhibited cathepsin B as a time-dependent irreversible inhibitor (Figure S1) with inactivation rate of 43.9±11.1 M^−1^s^−1^ (Table 2). However, in the presence of 5 mM cysteine no such irreversible inhibition was observed during the time course of the experiment (Figure S2).

### Cathepsin inactivation by 4-aminophenols is ROS dependent

**Figure 3:**
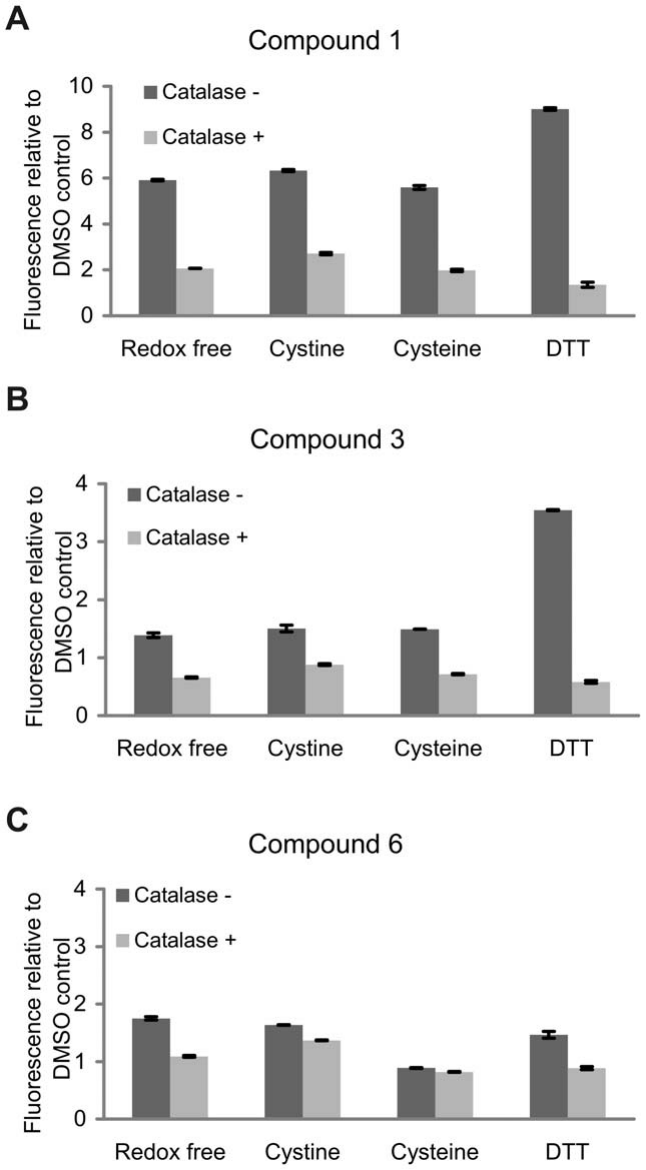
4-Aminophenols induce formation of ROS in various redox systems as shown by the fluorescent probe H_2_DCFDA. 10 µL of the de-esterified probe (see Methods) and 5 µl of DMSO (control) or compound **1** (A), **3** (B) and **6** (C) (500 µM) were added to the wells of a black microplate and after addition of 85 µl of activation buffer with a corresponding redox agent the reaction was monitored at 535 nm excitation and 485 nm emission wavelengths for 15 min at 37°C. In a parallel experiment catalase (200 U/ml) was added to the activation buffer. All measurements were performed in duplicate and the data are representative of three independent experiments.

To establish whether ROS induced by 4-aminophenols are mediators of time-dependent irreversible inactivation of cathepsins, a probe, H_2_DCFDA, was used. The latter upon de-esterification and oxidation becomes fluorescent and is used for ROS detection. In the presence of compound **1** an approximately 6–fold greater production of ROS was observed in comparison with DMSO control when redox-free, 5 mM cystine and 5 mM cysteine conditions were used (Figure 3A). A significantly higher response (9.0–fold increase) was obtained with 5 mM DTT as reducing agent in the activation buffer. The implication that H_2_O_2_ is one of the ROS induced by compound **1** was corroborated by the addition of catalase, which degrades H_2_O_2_ to O_2_ and H_2_O. Catalase significantly abrogated the ROS signal under all redox conditions used, confirming H_2_O_2_ as one of the ROS induced by compound **1**. When compound **3** was evaluated for ROS production a similar, albeit less pronounced, trend was observed (Figure 3B). The ROS signal was again higher with 5 mM DTT used as a reducing agent (3.5–fold increase) whereas the increase of the ROS signal under redox-free, cystine and cysteine conditions was smaller (∼1.5–fold increase). Upon addition of catalase, a significant reduction in H_2_DCFDA signal was evident, as was the case with compound **1**. For compound **6** the increase in ROS signal was only minor −1.7, 1.6 and 1.5–fold increases for redox-free, 5 mM cystine and 5 mM DTT conditions (Figure 3C). Furthermore, compound **6** did not induce ROS production in the presence of 5 mM cysteine. However, addition of catalase to the assay buffer significantly decreased the ROS signal under redox-free, 5 mM cystine and 5 mM DTT conditions. These results indicate that compounds **1** and **3** are capable of inducing ROS production in various redox systems with the most pronounced production in the presence of DTT. Compound **6** is also capable of induction of ROS, although to a lesser extent.

### Cathepsin B inactivation by 4-aminophenols is active site directed

**Figure 4:**
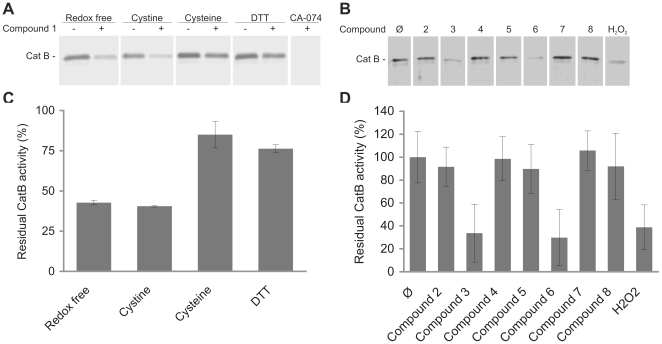
Compound 1 and its analogues (compounds 2–8) inactivate cathepsin B through an active site directed mechanism. Cathepsin B (1 µg) was incubated with compound **1** (200 µM) in activation buffers containing various redox agents at 5 mM (A, C) or with compounds **2**–**8** and H_2_O_2_ (200 µM) in the activation buffer in the absence of redox compounds (B, D) and subsequently labeled with DCG-04 (2 µM). Samples were then analyzed by SDS-PAGE and Western blotting. The band intensities indicate cathepsin B activity. The graphs of residual cathepsin B activity were obtained by dividing individual band intensities with that of the suitable control (C, D). Data shown here are presented as means ± SD, n = 2.

To determine whether the inactivation of cathepsin B by 4-aminophenols is due to the oxidation of the active site thiol rather than other cysteine residues we performed three independent experiments. The first was based on the use of the active site probe DCG-04 [29] which binds irreversibly into the active site of cysteine cathepsins and can be detected through a biotin tag, the intensity of labeling correlating with protease activity. In the first instance, compound **1** was tested in the presence of various redox systems. In non-reducing environments (redox-free and 5 mM cystine) compound **1** inhibited cathepsin B activity by 60% (Figure 4A and C). In the presence of 5 mM cysteine and 5 mM DTT the inhibition was reduced to 15% and 25%. Cathepsin B activity was completely inhibited by the specific irreversible inhibitor CA-074, which was used as a positive control. Binding of CA-074 into the active site of the enzyme was not affected by the redox environment as shown in Figure S3. Compounds **2**–**8** and H_2_O_2_ were also tested for their ability to prevent DCG-04 from binding into the active site cleft. In the redox-free system only compounds **3**, **6** and H_2_O_2_ inhibited cathepsin B activity – by 66, 70 and 60%, respectively (Figure 4B and D). No significant change in cathepsin B activity was seen with compounds **2**, **4**, **5**, **7** and **8**. However, when 5 mM cysteine was used as a reducing agent, compounds **2–8** did not significantly alter DCG-04 binding and, of **3**, **6** and H_2_O_2_, only compound **3** displayed inhibition, at 32% (Figure S4).

**Figure 5:**
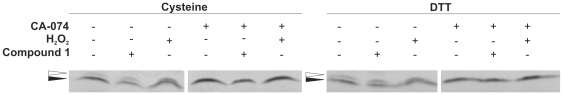
Compound 1 induces oxidative changes in the active site of cathepsin B. Cathepsin B (2 µg) was incubated with DMSO or CA-074 (50 µM) for 15 min at RT in activation buffer with respective reducing agent at 5 mM. Afterwards, the samples were incubated with DMSO, compound **1** (500 µM) or H_2_O_2_ (490 µM) for 5 h at RT. 4 µl of each sample was then loaded on precast isoelectric focusing gel (pI range 3.0–9.0), separated on the Phast System and stained with Coomassie blue. Black and white arrows are used to denote the active and the oxidized isoforms of cathepsin B, respectively.

In a second experiment, redox transformations of the active site thiol group to either sulfenic, sulfinic or sulfonic acid were demonstrated through changes in pI of the enzyme using IEF. Recombinant cathepsin B, expressed in *E. coli*, was found to consist of 2 isoforms with pI values of 5.46 and 5.35 (Figure 5). These pI values are close to the predicted pI value for the single chain form of the enzyme (5.24) [30] and are in line with pI values for human liver cathepsin B (4.6–5.2) [31] and for cathepsin B isolated from human osteosarcoma cell line (5.5–5.9) [32]. The isoform with the pI value of 5.46 corresponds to the active form, whereas the band at the pI value of 5.35 can be attributed to the oxidized enzyme form, since addition of H_2_O_2_ as a positive control induced a shift from the active to the oxidized isoform. Compound **1** also induced the oxidized form of cathepsin B. The ratio between the active and the oxidized form of cathepsin B was determined with scanning and quantification of band intensities. DMSO-treated cathepsin B displayed 1∶0.60 and 1∶0.61 ratio between the active and the oxidized form of cathepsin B in the presence of 5 mM cysteine and DTT, respectively. Treatment with compound **1** changed the ratio in favor of the oxidized form to 1∶0.79 and 1∶0.91, respectively. Additionally, samples were pretreated with CA-074 which binds irreversibly into the active site cleft, thus preventing oxidation of the active site thiol by compound **1** or by H_2_O_2_. Hence, the pretreatment caused the oxidized isoform to disappear and only one band was visible, in this way confirming the oxidation of the active site thiol of cathepsin B by compound **1**.

**Figure 6:**
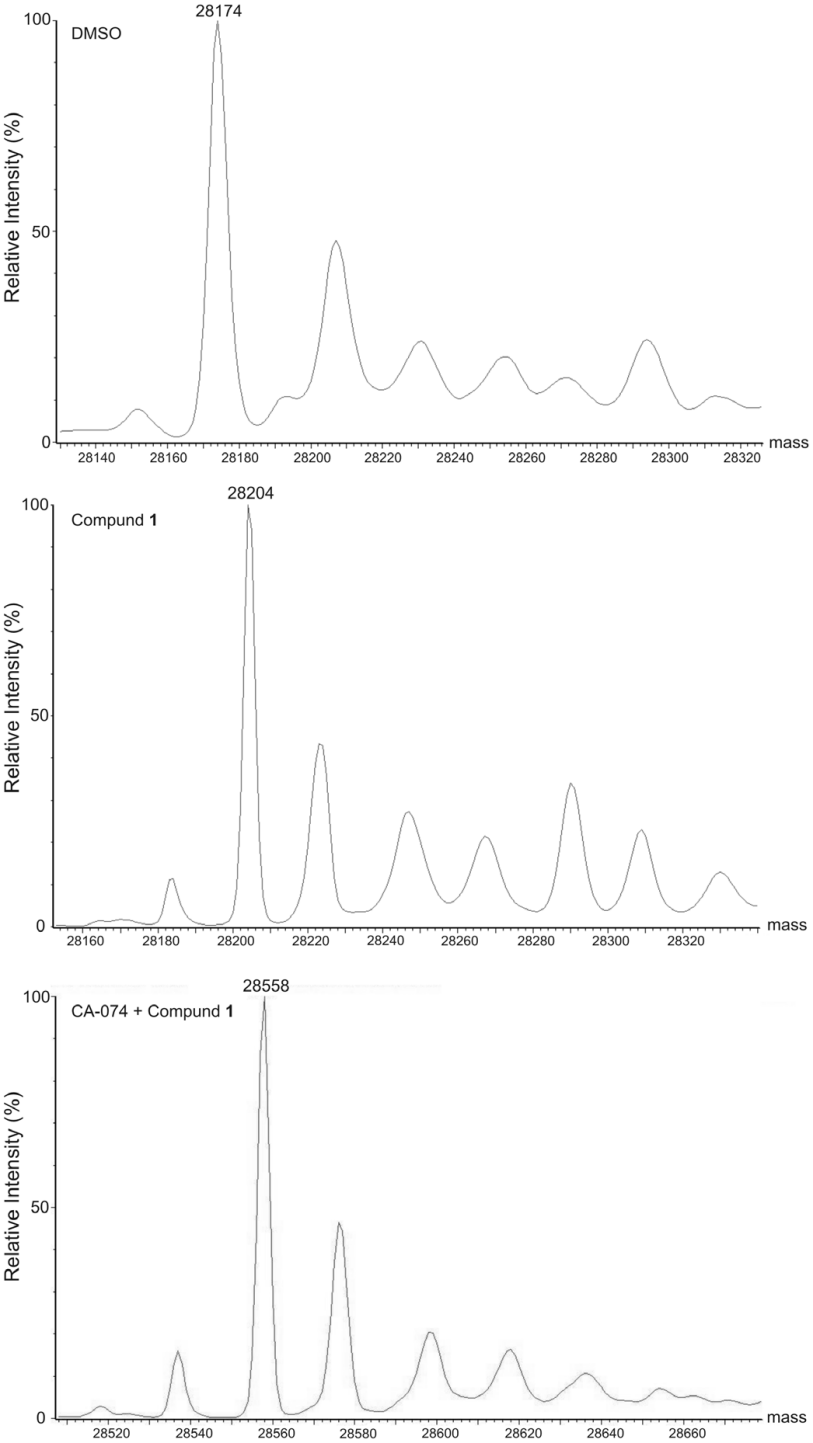
Identification of compound 1-induced cathepsin B modification by mass spectrometry. Cathepsin B (4.4 µM) was treated with DMSO or CA-074 (44 µM) and after 1 h compound **1** (500 µM) or DMSO were added and incubated for additional 5 h at RT. The mixtures were then dialyzed against water and analyzed by ESI-MS.

Additionally, mass spectrometry was used to identify the compound **1**-induced protein modification of cathepsin B. Mass spectrometry analysis of DMSO-treated cathepsin B resulted in one major peak with mass of 28174±1 Da (Figure 6). After 5 h incubation with compound **1** (500 µM) the peak shifted to a mass of 28204±1 Da, consistent with the addition of two oxygen atoms and formation of sulfinic acid. To show that this modification is active-site directed cathepsin B was treated with CA-074 (44 µM) prior to addition of compound **1**. This resulted in an increase in mass of 28558±1 Da, corresponding to the formation of the covalent adduct between cathepsin B and CA-074 (calculated mass of bound fragment: 384.21 Da). CA-074 is known to covalently react only with the active site thiol group [10] leaving other thiol groups free for modification. However, no peaks were evident that could be attributed to CA-074-cathepsin B covalent adducts with two additional oxygen atoms. Thus, compound **1** induces formation of sulfinic acid from the active site thiol group under the experimental conditions used.

## Discussion

These results demonstrate that 4-aminophenols inactivate cysteine cathepsins B, H and L in a dose- and time-dependent manner. The inhibition is mediated by ROS, of which H_2_O_2_ is shown to be a major constituent. The inactivation of cysteine cathepsins arises from specific oxidation of the thiol group of the catalytic Cys29 to sulfinic acid.

Cysteine cathepsins are small monomeric enzymes with molecular weight of approximately 30 kDa and possess a papain-like structure consisting of a left and a right domain. The two domains form a V-shaped active site cleft in the middle of which the catalytic triad is located [33]. The catalytic Cys25 (papain numbering) possesses an unusually low pK_a_ (∼ 2.5–3.5) [34] for a cysteine residue and is in the form of a thiolate which forms a thiolate-imidazolium ion pair with His159, located on the opposite site of the active site cleft. The low pK_a_ of the thiolate enhances its catalytic function as a nucleophile, however, it also increases its susceptibility to inactivation by ROS, such as H_2_O_2_ [35]. To avoid false positive inhibition by RCCs during the search for new cathepsin inhibitors a simple assay that could distinguish nuisance redox compounds from well-behaved inhibitors would be useful. Two high throughput plate-based assays have recently been reported. The first detects small molecule redox activity through oxidative transformation of resazurin to resorufin [21] while, in the second, H_2_O_2_ generated by RCCs is detected with horseradish peroxidase mediated oxidation of phenol red [36]. Using the latter, Soares et al. screened the National Institutes of Health Small Molecule Repository library for RCCs, and eight different structural classes of RCCs – *ortho*-quinones, *para*-quinones, pyrimidotriazinediones, arylsulfonamides, nitrothiophene-2-carboxylates, tolyl hydrazides, acetamido-dihydroisoxazolopyridine-diyl diacetates and pyrrolo-quinoxalinchlorobenzenamides were revealed [22]. Of the reported structural classes the quinone group appears to be the best characterized as they are reported to be redox-based inactivators of caspase-3 [20], caspase-8 [18], protein tyrosine phosphatase 1B [19] and protein tyrosine phosphatase α [17]. Here, we report a novel small-molecule scaffold capable of inducing oxidative inactivation of cysteine cathepsins through ROS generation.

As part of a previous study we discovered that compound **1** inhibits cathepsin B in a time-dependent manner. Given that it lacks an electrophilic motif, typically required for irreversible inactivation of the enzyme, and that time-dependent behavior is one of the first indicators of RCCs [16], redox-based inactivation of cathepsin B through ROS generation was suspected. Compound **1** is seen to be a potent irreversible inhibitor of cathepsin B in redox-free and cystine systems (Table 1). The second-order rate constants are comparable with those reported for nonspecific redox inactivation of protein tyrosine phosphatase α by *ortho*-quinones (67.8 M^−1^s^−1^) [17]. Under reducing conditions the inactivation by compound **1** was significantly attenuated. These results contrast with reports that redox inactivation by RCCs requires the presence of strong reducing agents like DTT or tris(2-carboxyethyl)phosphine (TCEP) and is not observed in the presence of milder reducing agents, such as glutathione, β-mercaptoethanol, cysteine, or in their absence [17]–[20]. However, in the latter studies, quinone-like compounds were used, which require a strong reducing agent like DTT to reduce the quinone moiety to hydroquinone which could then undergo synproportionation with a quinone molecule and enter the redox cycle [16]. Our results thus establish that 4-aminophenols are capable of entering the redox cycle without intervention of a reducing agent.

Since RCCs are known for their promiscuous bioactivity profiles and can inhibit different classes of enzymes, such as protein tyrosine phosphatases and cysteine proteases (cathepsins and caspases) [16], we sought to discover whether compound **1** is able to distinguish between individual cathepsins. Cathepsins B, H and L were chosen based on their substrate specificities. Cathepsin H acts as an aminopeptidase, cathepsin L as an endopeptidase and cathepsin B can cleave its substrates both as an endopeptidase and a dipeptidyl carboxypeptidase [37]. Compound **1** inhibited all three enzymes within the same concentration range (Table 1) in non-reducing and reducing conditions, with no indication of inter-cathepsin specificity.

A series of compound **1** analogues (compounds **2**–**8**) were also tested for the inhibition of cathepsin B (Figure S1 and S2) to elucidate which structural features are responsible for the time-dependent inactivation. Of the entire analogue series only compounds **1**, **3** and **6** displayed time-dependent inactivation in the non-reducing environment (Table 2) leading to the conclusion that the time-dependent irreversible inactivation of cathepsin B requires the complete 4-aminophenol moiety. As is evident from compound **3** the quinoline nitrogen is not a prerequisite for enzyme inactivation. On the contrary, compound **3** inhibited cathepsin B 12-fold and 42-fold better than compound **1** under non-reducing and reducing conditions. Furthermore, the aromatic ring in compounds **1** and **3** is not essential for cathepsin B inactivation under redox-free conditions since compound **6**, lacking the quinoline or naphthalene core, retains its inhibitory activity against cathepsin B. However, both amino and hydroxyl group in the *para* position are essential for cathepsin B inactivation since compound **7** lacking the amino group or compound **8** lacking the hydroxyl group lose their time-dependent inactivation. Of the different aminophenols (2-, 3- and 4-aminophenol), only 4-aminophenol was able to non-enzymatically induce hydroxyl radical formation [38], therefore, we did not examine the 2- and 3- substituted aminophenols for cathepsin B inhibition.

Of the three inhibitors (compounds **1**, **3** and **6**) that behaved in a time-dependent manner in the non-reducing environment, only compounds **1** and **3** retained their time-dependent inhibition in a reducing environment (Figure S2). Although compound **6** was still able to inhibit cathepsin B, it did not exhibit pseudo-first order inactivation kinetics, but behaved as a reversible inhibitor. Similarly, H_2_O_2_ also behaved as a time-dependent inhibitor under non-reducing conditions (Table 2). The value, (k_inact_/K_I_ of 43.9±11.1 M^−1^s^−1^) is in line with those for the H_2_O_2_ induced inactivation of the related cysteine protease papain (k_inact_/K_I_ of 61.7 M^−1^s^−1^) [35] and protein tyrosine phosphatases (k_inact_/K_I_ of 10–20 M^−1^s^−1^) [39]. However, in the presence of 5 mM cysteine, it inhibits cathepsin B as a reversible inhibitor. These results indicate H_2_O_2_ as one of the components of ROS induced by compound **6**. H_2_O_2_ induces oxidation of the papain active site thiol group to inactive sulfenic acid in the absence of reducing agents, a step which is almost completely reversed by addition of cysteine [35]. Additionally, protein tyrosine phosphatases react with H_2_O_2_, yielding the sulfenic acid of the active site cysteine, which can be reduced back to the active thiolate species with glutathione, DTT, β-mercaptoethanol and cysteine [39].

4-aminophenols were shown to induce formation of ROS in various redox environments in the presence of atmospheric oxygen using the fluorescent probe H_2_DCFDA (Figure 3). The increase in ROS production was most pronounced with compound **1**, followed by compound **3** and compound **6**. Moreover, addition of catalase to the activation buffer significantly abrogated induction of ROS signal by compounds **1** and **3** suggesting H_2_O_2_ as one of the components of induced ROS [16]. Although, H_2_DCFDA is known to have low reactivity towards H_2_O_2_, the latter can serve as a source of additional ROS, such as hydroxyl radical, which is readily detected with H_2_DCFDA probe [40].

Cathepsin B possesses 14 cysteine residues, of which 12 are involved in the formation of disulphide bonds, Cys29 is part of the catalytic triad and Cys240 is unpaired [41]. The pK_a_ of sulfhydryl groups of most cysteine residues in proteins is approximately 8.5 and they do not react at physiologically significant rates with H_2_O_2_ unless the reaction is catalyzed [16]. On the other hand, the active site cysteine of cathepsin B exists as a thiolate anion with a much lower pK_a_ rendering it more susceptible to oxidative inactivation. Compound **1** prevented DCG-04 from binding into the active site in reducing and non-reducing environments, confirming the active site directed inactivation (Figure 4). In addition, compounds **3** and **6,** as well as H_2_O_2_, abolished DCG-04 binding in a redox-free environment, suggesting a common active site directed mechanism of cathepsin inactivation for all 4-aminophenols. The result was confirmed by IEF, where addition of compound **1** resulted in increased intensity of a band corresponding to an oxidized form of cathepsin B. The latter completely disappeared after pretreatment with CA-074 (Figure 5), a cathepsin B specific inhibitor binding irreversibly to the active site cysteine, thereby preventing its oxidation. Additionally, mass spectrometry analysis was used to identify the modifications of cathepsin B by compound **1**. Treatment of cathepsin B with compound **1** induced formation of sulfinic acid and pretreatment with CA-074 prevented redox-changes to the enzyme confirming that inactivation of cathepsin B was active site-directed (Figure 6). Active site-directed inactivation also appears to be important for other reported classes of RCCs. A recent study reported the oxidative transformation of caspase-3 catalytic cysteine to sulfonic acid by isoquinoline-1,3,4-trione derivatives [20]. Two independent groups have also shown the transformation of the active site thiol group of cathepsin L and protein tyrosine phosphatase 1B to sulfinic and to sulfonic acid by RCCs [19], [21].

**Figure 7:**
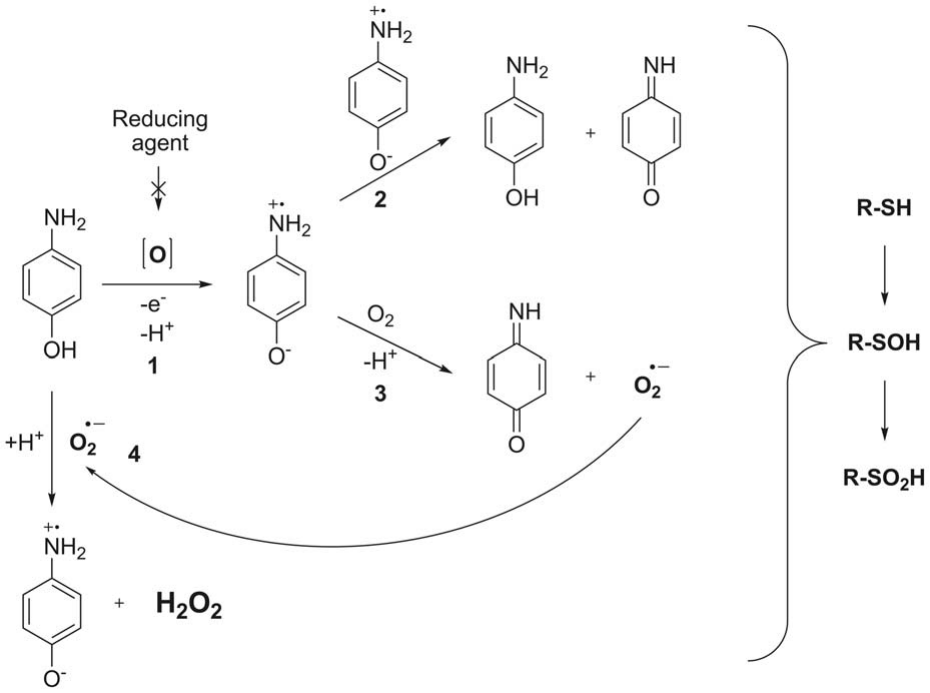
Schematic representation of 4-aminophenol induced ROS formation.

From these results we can predict the following mechanism for 4-aminophenol induced inactivation of cathepsins (Figure 7). 4-Aminophenol is light sensitive and undergoes non-enzymatic oxidation in aqueous solutions in the presence of molecular oxygen to form the 4-aminophenoxy radical [38], [42]–[44]. The 4-aminophenoxy radical then either undergoes disproportionation to yield the *p*-benzoquinoneimine or reacts with O_2_ to form the superoxide anion and the quinoneimine species [42]. The superoxide anion can then react with 4-aminophenol and generate H_2_O_2_ and the 4-aminophenoxy radical [17], thus completing the redox cycle. This proposed mechanism of redox cycling explains the lack of need for strong reducing agents like DTT and TCEP, as seen with quinone-like RCCs [16]. Moreover, it also explains why addition of the reducing agents used in this study diminishes the inactivation rates by 10–fold from those in non-reducing environments. They appear to protect 4-aminophenol from oxidation by air, as was reported for hydrazine and antioxidants such as ascorbate, glutathione and NADPH [45], [46]. The resulting ROS readily react with the active site thiolate anion, forming in the first step a sulfenic acid of cysteine (−SOH) which is fully reducible back to cysteine in the presence of reducing agents, unlike sulfinic acid (−SO_2_H), which is formed form upon further oxidation of sulfenic acid in the second step [39].

However, irreversible inactivation of cathepsins by 4-aminophenols can also be explained by a mechanism involving the formation of covalent adducts of *p*-benzoquinoneimines with the active site thiol group. This mechanism was proposed to be the main source of hepatotoxicity of acetaminophen. Its metabolic activation leads to the formation of a toxic metabolite, *N*-acetyl-*p*-benzoquinoneimine. The latter is preferentially conjugated with glutathione, but once glutathione is depleted the toxic metabolite binds to other nucleophilic groups in the cell, in this way accounting for the toxicity [47]. Nonetheless, the attenuation of ROS induction by catalase and of cathepsin inhibition in the presence of various reducing agents as well as mass spectrometry data support our hypothesis that 4-aminophenols inactivate cysteine cathepsins through oxidative inactivation. A recent study has also shown that 4-aminophenol induces H_2_O_2_ formation in cell medium, impairing LLC-PK_1_ cell viability, in this way exerting its toxicity [44].

To conclude, we report 4-aminophenols as a novel class of RCCs capable of entering the redox cycle, thereby inducing the formation of ROS and producing false positives in enzyme inhibition assays. We propose that compounds with a 4-aminophenol scaffold are eliminated from compound libraries to be screened for inhibition of cysteine proteases. This could be of great importance in HTS campaigns, where the exclusion of false positive hits saves both time and resources otherwise spent on follow up studies.

## Supporting Information

Figure S1 **Progress curves of cathepsin B activity obtained in the presence of 4-aminophenol analogues in redox-free conditions.** 5 µl of DMSO (control) or compounds **1** (A), **2** (B), **3** (C), **4** (D), **5** (E), **6** (F), **7** (G), **8** (H) and H_2_O_2_ (I) (fifteen concentrations were used for each experiment, for clarity only six are shown) and 5 µl of Z-Arg-Arg-AMC (60 µM) were added to wells of a black microplate. Reaction was initiated by adding 90 µl of cathepsin B (400 pM). Formation of fluorescent degradation product was monitored continuously at 380 nm excitation and 460 nm emission wavelengths using the minimal kinetic interval at 37°C. All kinetic measurements were performed in duplicate. For time-dependent inhibitors each progress curve was fitted to the equation *P  =  v_s_t + (v_i_ − v_s_)(1 − e^−kobst^)/k_obs_*. The obtained *k_obs_* were plotted against inhibitor concentration and the slope of linear fit yielded the second-order rate constant *k_inact_/K_I_*.
(TIF)

Figure S2 **Progress curves of cathepsin B activity obtained in the presence of 4-aminophenol analogues in the presence of 5 mM cysteine.** 5 µl of DMSO (control) or compounds **1** (A), **2** (B), **3** (C), **4** (D), **5** (E), **6** (F), **7** (G), **8** (H) and H_2_O_2_ (I) (fifteen concentrations were used for each experiment, for clarity only six are shown) and 5 µl of Z-Arg-Arg-AMC (60 µM) were added to the wells of a black microplate. The reaction was initiated by adding 90 µl of cathepsin B (400 pM). Formation of fluorescent degradation product was monitored continuously at 380 nm excitation and 460 nm emission wavelengths using the minimal kinetic interval at 37°C. All kinetic measurements were performed in duplicate. For time-dependent inhibitors each progress curve was fitted to the equation *P  =  v_s_t + (v_i_ − v_s_)(1 − e^−kobst^)/k_obs_*. The obtained *k_obs_* were plotted against inhibitor concentration and the slope of linear fit yielded the second-order rate constant *k_inact_/K_I_*.
(TIF)

Figure S3 **Binding of CA-074 is not affected by the redox environment.** Cathepsin B (1 µg) was incubated with cathepsin B specific irreversible inhibitor CA-074 (200 µM) in a variety of activation buffers containing no reducing agent, cystine, cysteine or DTT (5 mM) and subsequently labeled with DCG-04 (2 µM). Samples were then analyzed by SDS-PAGE and Western blotting. The band intensities indicate cathepsin B activity.
(TIF)

Figure S4 **Compounds 2–8 and H_2_O_2_ do not significantly impair cathepsin B activity in the presence of 5 mM cysteine, as shown with the DCG-04 probe.** Cathepsin B (1 µg) was incubated with compounds **2**–**8** and H_2_O_2_ (200 µM) in the activation buffer containing 5 mM cysteine (A) and subsequently labeled with DCG-04 (2 µM). Samples were then analyzed with SDS-PAGE and Western blotting. Band intensities correlate with cathepsin B activity. Residual cathepsin B activity was obtained by dividing individual band intensity by that of the control (B). Data shown here are presented as means ± SD, n = 2.
(TIF)

Figure S5 **pI calibration curve.** The latter was obtained using the Broad range pI calibration kit (pH 3.0–10.0) according to the protocol of the manufacturer.
(TIF)
