# Supplementary figures and images for: Redox-Based Inactivation of Cysteine Cathepsins by Compounds Containing the 4-Aminophenol Moiety
Source: PLoS One. 2011 Nov 4;6(11):e27197. doi: 10.1371/journal.pone.0027197 (PMC3208577; doi:10.1371/journal.pone.0027197)

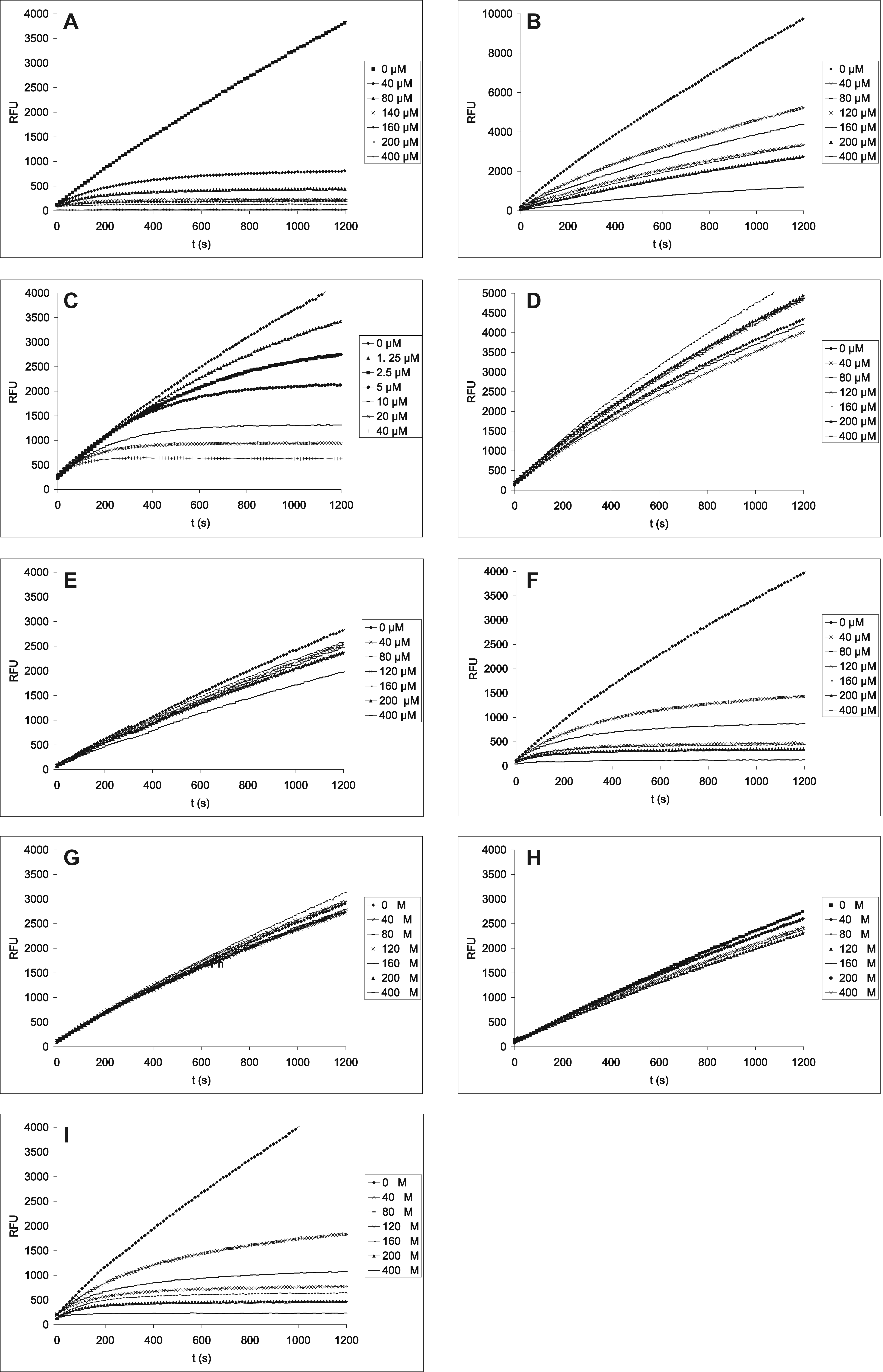

Supplement: Figure S1 — Progress curves of cathepsin B activity obtained in the presence of 4-aminophenol analogues in redox-free conditions. 5 µl of DMSO (control) or compounds 1 (A), 2 (B), 3 (C), 4 (D), 5 (E), 6 (F), 7 (G), 8 (H) and H2O2 (I) (fifteen concentrations were used for each experiment, for clarity only six are shown) and 5 µl of Z-Arg-Arg-AMC (60 µM) were added to wells of a black microplate. Reaction was initiated by adding 90 µl of cathepsin B (400 pM). Formation of fluorescent degradation product was monitored continuously at 380 nm excitation and 460 nm emission wavelengths using the minimal kinetic interval at 37°C. All kinetic measurements were performed in duplicate. For time-dependent inhibitors each progress curve was fitted to the equation P = vst + (vi − vs)(1 − e−kobst)/kobs. The obtained kobs were plotted against inhibitor concentration and the slope of linear fit yielded the second-order rate constant kinact/KI. (TIF) [file pone.0027197.s001.tif]

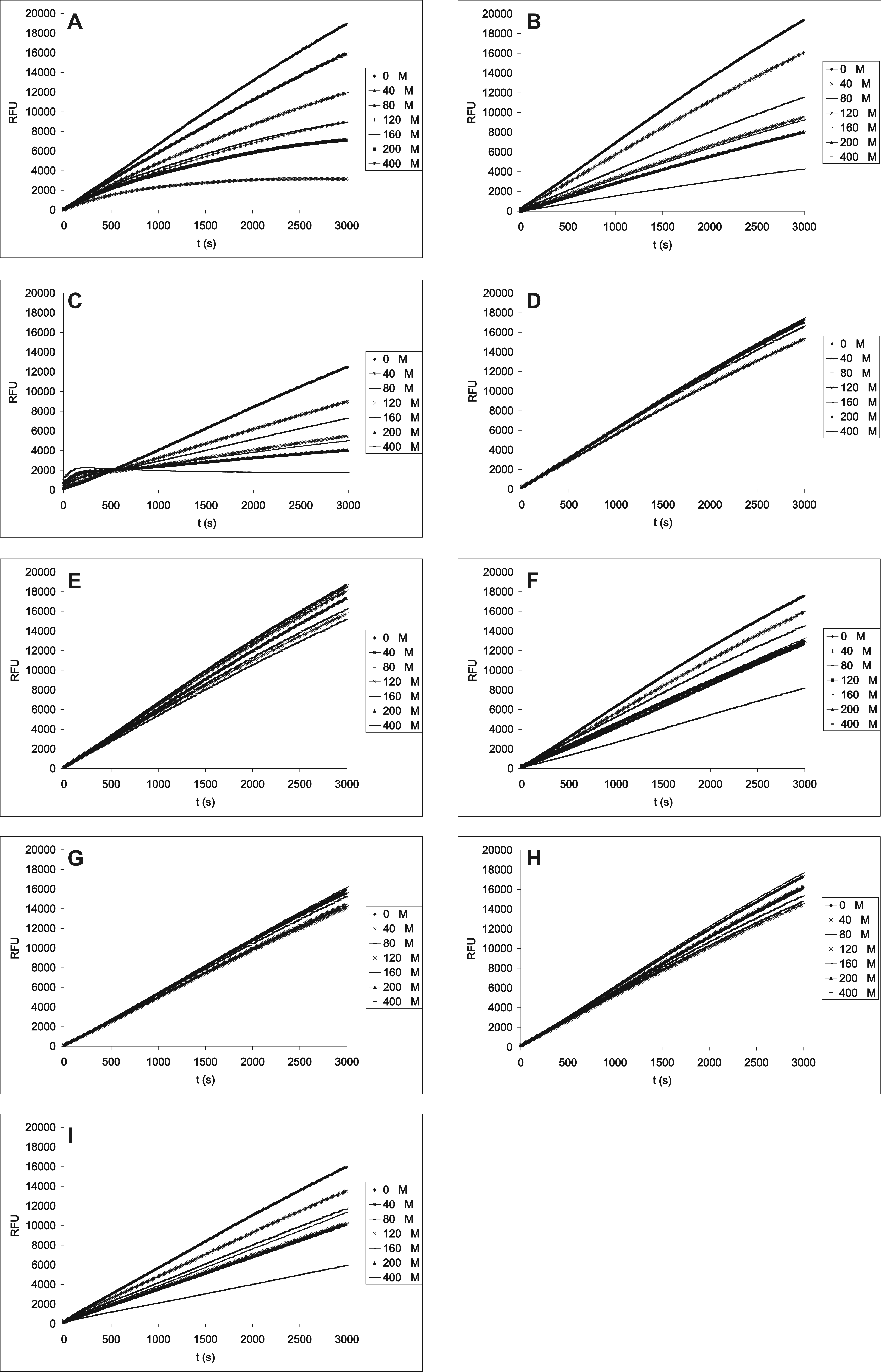

Supplement: Figure S2 — Progress curves of cathepsin B activity obtained in the presence of 4-aminophenol analogues in the presence of 5 mM cysteine. 5 µl of DMSO (control) or compounds 1 (A), 2 (B), 3 (C), 4 (D), 5 (E), 6 (F), 7 (G), 8 (H) and H2O2 (I) (fifteen concentrations were used for each experiment, for clarity only six are shown) and 5 µl of Z-Arg-Arg-AMC (60 µM) were added to the wells of a black microplate. The reaction was initiated by adding 90 µl of cathepsin B (400 pM). Formation of fluorescent degradation product was monitored continuously at 380 nm excitation and 460 nm emission wavelengths using the minimal kinetic interval at 37°C. All kinetic measurements were performed in duplicate. For time-dependent inhibitors each progress curve was fitted to the equation P = vst + (vi − vs)(1 − e−kobst)/kobs. The obtained kobs were plotted against inhibitor concentration and the slope of linear fit yielded the second-order rate constant kinact/KI. (TIF) [file pone.0027197.s002.tif]

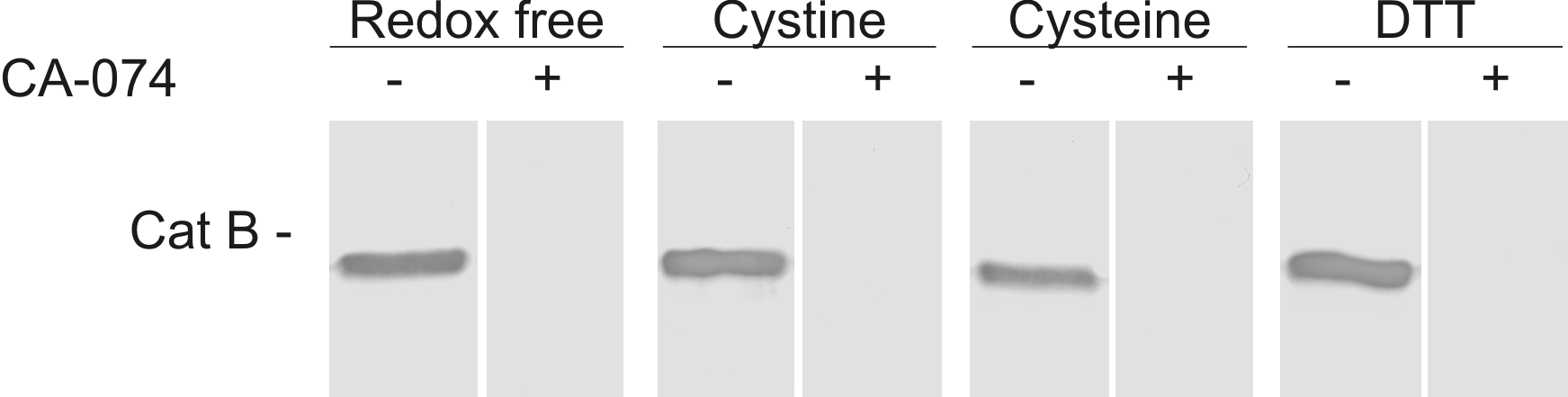

Supplement: Figure S3 — Binding of CA-074 is not affected by the redox environment. Cathepsin B (1 µg) was incubated with cathepsin B specific irreversible inhibitor CA-074 (200 µM) in a variety of activation buffers containing no reducing agent, cystine, cysteine or DTT (5 mM) and subsequently labeled with DCG-04 (2 µM). Samples were then analyzed by SDS-PAGE and Western blotting. The band intensities indicate cathepsin B activity. (TIF) [file pone.0027197.s003.tif]

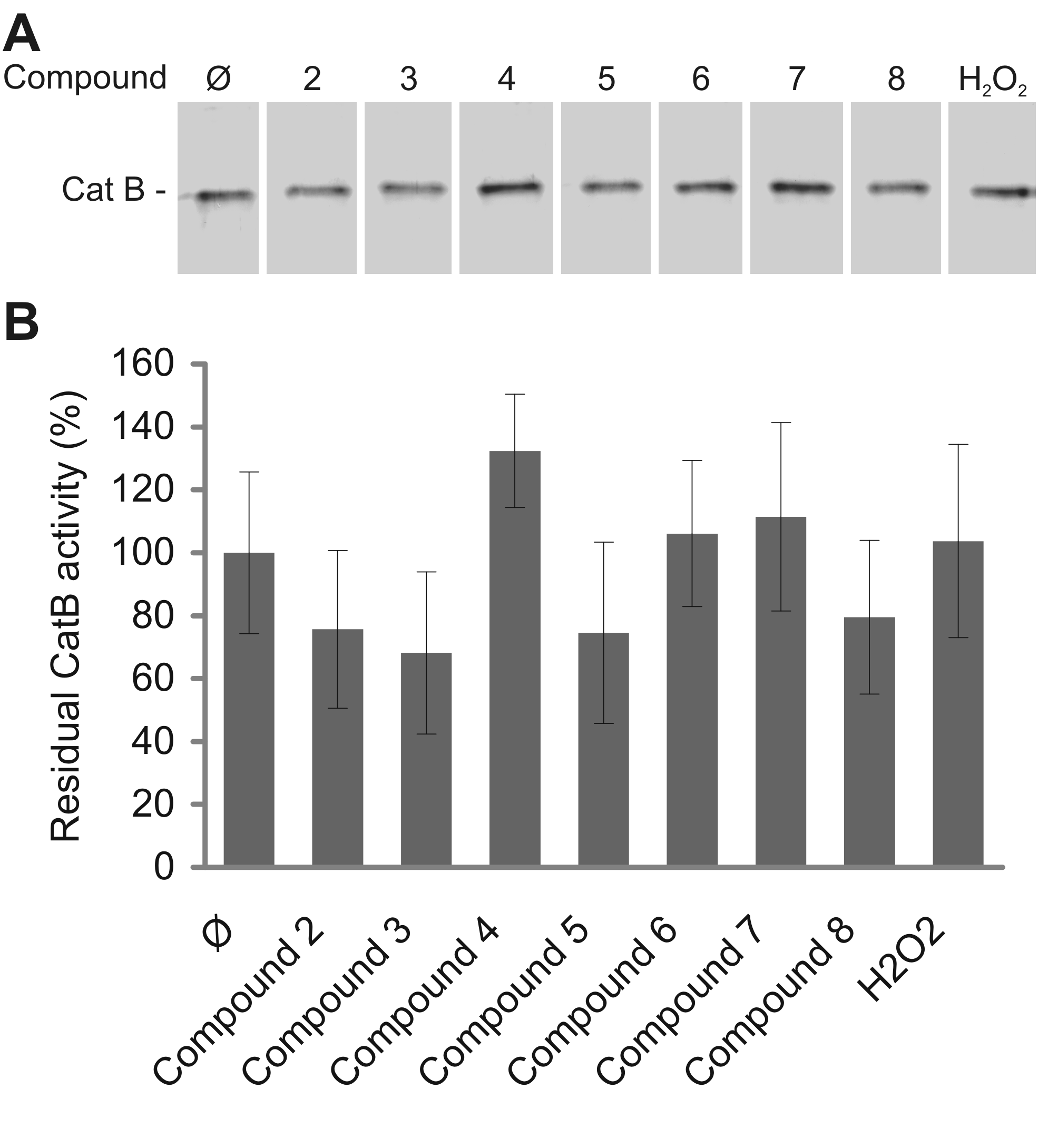

Supplement: Figure S4 — Compounds 2–8 and H2O2 do not significantly impair cathepsin B activity in the presence of 5 mM cysteine, as shown with the DCG-04 probe. Cathepsin B (1 µg) was incubated with compounds 2–8 and H2O2 (200 µM) in the activation buffer containing 5 mM cysteine (A) and subsequently labeled with DCG-04 (2 µM). Samples were then analyzed with SDS-PAGE and Western blotting. Band intensities correlate with cathepsin B activity. Residual cathepsin B activity was obtained by dividing individual band intensity by that of the control (B). Data shown here are presented as means ± SD, n = 2. (TIF) [file pone.0027197.s004.tif]

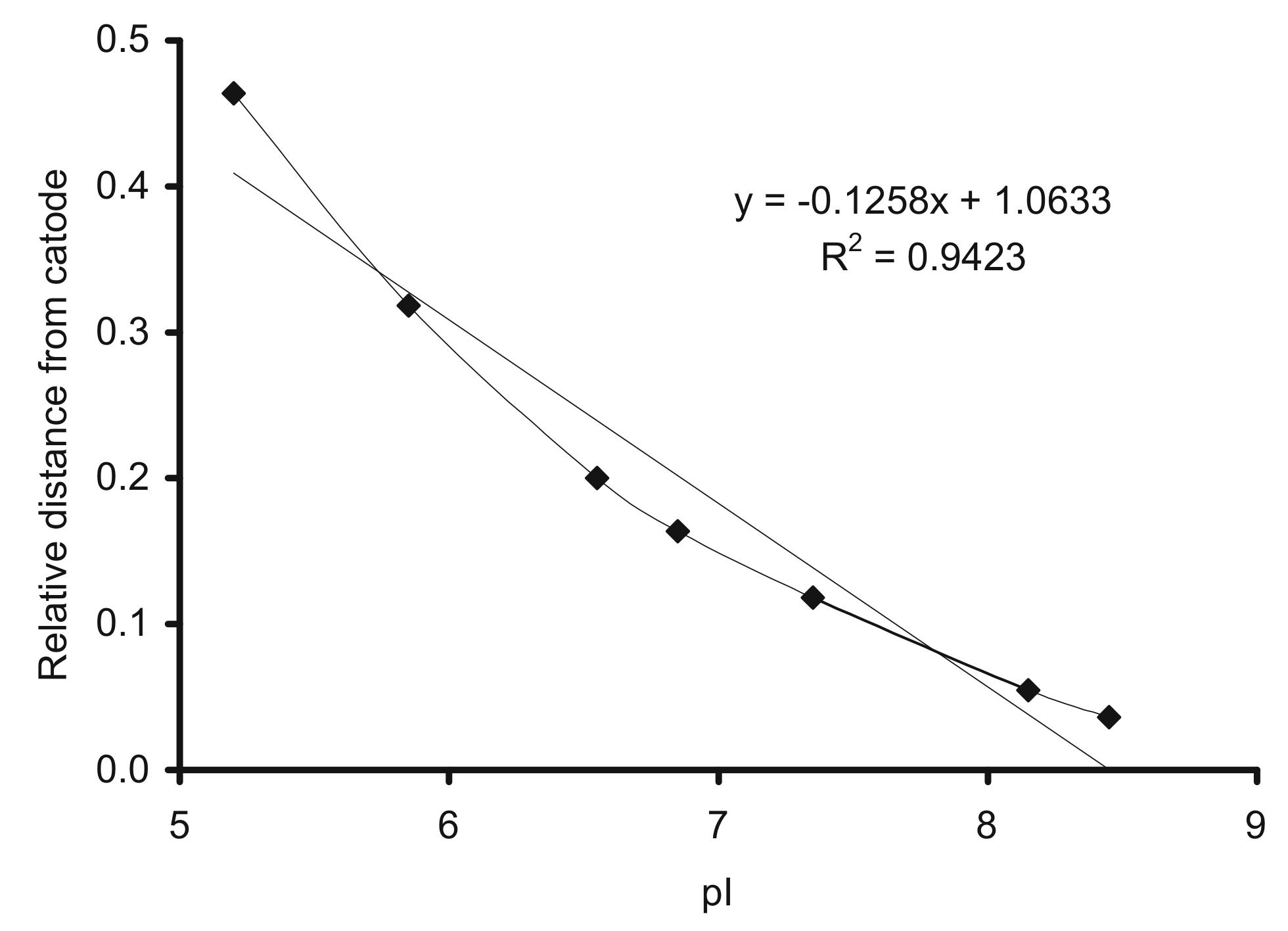

Supplement: Figure S5 — pI calibration curve. The latter was obtained using the Broad range pI calibration kit (pH 3.0–10.0) according to the protocol of the manufacturer. (TIF) [file pone.0027197.s005.tif]
